# A systematic review and meta-analysis of association between brain-derived neurotrophic factor and type 2 diabetes and glycemic profile

**DOI:** 10.1038/s41598-021-93271-z

**Published:** 2021-07-02

**Authors:** Milad Davarpanah, Nafiseh Shokri-mashhadi, Rahele Ziaei, Parvane Saneei

**Affiliations:** 1 Students’ Research Committee, Isfahan University of Medical Sciences, Isfahan, Iran; 2 Department of Community Nutrition, School of Nutrition and Food Science, Food Security Research Center, Isfahan University of Medical Sciences, PO Box 81745-151, Isfahan, Iran; 3 Department of Clinical Nutrition, School of Nutrition and Food Science, Food Security Research Center, Isfahan University of Medical Sciences, Isfahan, Iran

**Keywords:** Neuroscience, Diseases

## Abstract

Several epidemiologic studies have evaluated the relation between serum/plasma brain-derived neurotrophic factor (BDNF) levels and glycemic parameters, but the findings were conflicting. We performed a systematic review and meta-analysis to compare circulating BDNF levels in individuals with type 2 diabetes (T2D) or other glycemic disorders with healthy controls and to evaluate correlation between BDNF concentrations with glycemic profile. A systematic search up to July 2020 was conducted in reliable electronic databases (MEDLINE (Pubmed), EMBASE, Scopus) and Google scholar. Sixteen observational studies compared serum/plasma BDNF levels in diabetic patients (or individuals with glycemic disorders) vs. healthy controls or reported correlations between serum BDNF levels and glycemic parameters in adults were included in the review. Overall weighted mean difference (WMD) of circulating BDNF levels in 1306 patients with T2D (or other glycemic disorders) was 1.12 ng/mL lower than 1250 healthy subjects (WMD: − 1.12; 95%CI − 1.37, − 0.88, I^2^ = 98.7%, P < 0.001). Subgroup analysis revealed that both diabetic patients and subjects with other glycemic disorders had lower serum/plasma BDNF levels than healthy controls (WMD: − 1.74; 95%CI − 2.15, − 1.33 and WMD: − 0.49; 95%CI − 0.82, − 0.16, respectively). No significant correlation was found between BDNF levels and glycemic parameters [fasting blood glucose (FBG) (Fisher’s Z = 0.05; 95%CI − 0.21, 0.11; n = 1400), homeostatic model assessment for insulin resistance (HOMA-IR) (Fisher’s Z = 0.12; 95%CI − 0.20, 0.44; n = 732) and glycosylated hemoglobin (HbA1c) (Fisher’s Z = 0.04; 95%CI − 0.05, 0.12; n = 2222)]. We found that diabetic patients and subjects with glycemic disorders had lower circulating BDNF levels than healthy controls. However, there was no significant correlation between BDNF concentrations and glycemic parameters including FBG, HOMA-IR and HbA1c. Further prospective investigations are required to confirm these findings.

## Introduction

The global prevalence of type 2 diabetes (T2D) has increased sharply during the past 40 years^1^. Glycemic parameters including fasting blood glucose (FBG), glycosylated hemoglobin (HbA1c) and oral glucose tolerance test (OGTT) are used for diagnosis and control of T2D^2^. It has been found that insulin resistance (IR) is the core defect in T2D and some other disorders such as metabolic syndrome (MetS) and obesity^3^. IR is a condition in which cells of muscle, fat and liver do not respond to insulin and do not take up glucose, despite high serum levels of insulin^4^. Insulin plays a vital role in both glucose and fatty acids metabolism. Although the gold standard for defining IR is hyperinsulinemic-euglycemic clamp, homeostatic model assessment for insulin resistance (HOMA-IR) is mostly used in clinical practice^4^.

Prior investigations have shown that a number of risk factors including genetic and environmental factors are associated with insulin resistance^5^. Physical activity/exercise and nutrition as modifiable factors can also play significant roles in development and progression of IR^5,6^. Recent studies reported that some biomarkers such as brain-derived neurotropic factor (BDNF) have correlated with IR^12^. BDNF is a nerve growth factor which is expressed in the central nervous system (CNS) and other tissues including liver, pancreas and adipose tissues^7^. BDNF is peripherally stored in platelets and released into plasma^8^. The identified function of BDNF is differentiation and maturation of neurons^9^. This biomarker is crucial in modulating memory-associated neuroplasticity by regulating cell survival, proliferation, and synaptic growth in the developing CNS^10^. BDNF could induce long-term potentiation, as the neurophysiological basis of learning and memory^11^. Some previous studies have reported that serum BDNF levels were significantly lower in patients with cognitive disorders, such as cognitive impairment^12^ and Alzheimer’s disease^13^. Several other investigations reported that T2D was associated with deficits in many domains of cognitive function, such as learning, executive function, processing speed, and immediate and delayed memory^14,15^. On the other hand, poor cognitive function could increase the risk of severe hypoglycemia in patients with T2D^16^. So, individuals with T2D might have higher risk for cognitive disorders, including Alzheimer’s disease and dementia^17^. Also, findings from some previous reports have shown a link between BDNF and metabolism (including glucose and lipid metabolism and energy expenditure)^18^. BDNF seems to be associated with several inflammatory diseases, such as T2D, metabolic syndrome and atherosclerosis^19^.

Over recent decades, several epidemiologic studies have evaluated the relationship between serum/plasma BDNF levels and T2D, but the findings were conflicting. Decreased serum BDNF levels have been seen in some investigations in diabetic people in comparison to healthy controls^7,20,21^, while some others did not confirm these results^22,23^. In addition, numerous studies examined the correlation between glycemic parameters including (FBG, HbA1c, HOMA-IR) and BDNF; some of them found positive correlations between HOMA-IR and serum BDNF levels^22,23^, while the others suggested an inverse relationship^20,24^. In addition, different results have been reported based on gender, as in a case–control study a positive correlation between serum HbA1c concentrations and serum BDNF levels was found in men, while the results in women were totally conflicting^25^. Since the findings in this regard were not consistent and as we know, there is no previous review summarized these reports, we performed a systematic review and meta-analysis of epidemiologic studies to compare serum/plasma BDNF levels in individuals with T2D or other glycemic disorders with healthy controls and to evaluate the association between circulating BDNF levels and glycemic parameters, as well.

## Materials and methods

### Search strategy

A search of published studies was performed on some reliable databases (including MEDLINE (https://www.ncbi.nlm.nih.gov/pubmed) (PubMed), SCOPUS (https://www.scopus.com), EMBASE (https://www.elsevier.com), and Google scholar (https://scholar.google.com) from the earlier time available to the end of July 2020 with no limitation in language and time. The following Medical Subject Headings (MESH) and non-MESH keywords were used in the search: (“Blood Glucose”[MESH] OR “Blood Glucose”[Title/Abstract] OR “Fasting Plasma Glucose”[Title/Abstract] OR FBG[Title/Abstract] OR Insulin [MESH] OR “Insulin Resistance”[MESH] OR “Insulin Resistance”[Title/Abstract] OR “Insulin Levels”[Title/Abstract] OR HOMA-IR[Title/Abstract] OR “Glycated Hemoglobin A”[MESH] OR “Glycated Hemoglobin A”[Title/Abstract] OR HbA1C[Title/Abstract] OR “Diabetes Mellitus”[MESH] OR “Diabetes Mellitus”[Title/Abstract] OR “Metabolic Syndrome”[MESH] OR “Metabolic Syndrome”[Title/Abstract]) AND (BDNF[Title/Abstract] OR BDNF[MESH] OR “Brain-derived neurotrophic factor”[MESH] OR “Brain-derived neurotrophic factor”[Title/Abstract]). Moreover, a manual search according to reference lists of more relevant investigations was conducted to avoid missing any relevant data. After removing duplicate articles, abstracts were carefully read to evaluate the relevance of remained papers. Preferred Reporting Items for Systematic Reviews and Meta-Analyses checklist was used in this study to improve the quality of reporting^26^. Furthermore, the study was registered at Prospero (http://www.crd.york.ac.uk/Prospero; no. CRD42020201528).

### Inclusion criteria

Studies with the following criteria were eligible to be included in the present review: (1) were observational studies; (2) compared serum/plasma BDNF levels in patients with T2D (or glycemic disorders) vs. healthy people; or reported correlations between serum/plasma BDNF levels and glycemic parameters (including FBG, HgA1c, HOMA-IR, IR) in cases with diabetic (or other glycemic disorders), in healthy individuals or in a combination of diabetic and healthy subjects; (3) included adult population. Investigations were not included if they: (1) were interventional and experimental studies; (2) were conducted on animals; (3) were conducted on women with gestational diabetes mellitus; (4) were review articles; (5) were classified as gray literatures, for instance, conference abstracts, government reports and theses.

### Exclusion criteria

Studies were excluded if they: (1) reported only mean (± SD) or range of serum/plasma BDNF values in diabetic cases or healthy controls; (2) reported P value for correlation between serum/plasma BDNF and glycemic parameters, but did not report the correlation values.

### Data extraction

Following data were independently extracted from included studies by two investigators (NSM and RZ): first authors last name, the year of publication, the study location, type of study, number of participants in each group, gender of participants, mean serum/plasma BDNF levels (± SD or SE), different BDNF matrices (serum versus plasma), participants condition (fasting versus non-fasting), duration of having T2D, correlations between serum BDNF levels and glycemic parameters, type of Pearson or Spearman correlations, health status of participants. It is worth noting that any discrepancies between two investigators has been checked and discussed by the supervisor of the study (PS).

### Assessment the quality of studies

In order to assess the quality of included studies, the Newcastle–Ottawa Scale (NOS) suggested for observational studies was used. Different quality items, including selection, comparability and outcome assessment, were evaluated. NOS system assigns a maximum score of 9 to each study. In the present analysis, each study was considered as a high quality if it reached a score above median; otherwise, it deemed to a low quality study. Details of quality assessment of included case–control and cross-sectional studies are respectively presented in Supplemental Tables 1 and 2.

### Statistical methods

The mean difference and its standard deviation (SD) of serum/plasma BDNF levels in cases with T2D or other glycemic disorders and healthy controls were used for the meta-analysis. The correlation coefficients (reported for serum/plasma BDNF levels and glycemic profile) were also used for a separate analysis in this study. Fisher’s Z and its Standard Error (SE) were calculated based on the reported correlation coefficients and sample sizes of the studies. A random effects model that takes between-study variation into account was used to calculate the overall effect size. Between-study heterogeneity was assessed using Cochran’s Q test and I-Squared (I^2^). I^2^ values of 25, 50 and 75% are respectively referred to as low, moderate, and high estimates. In case of significant between-study heterogeneity, the sources of heterogeneity were detected by subgroup analysis and meta-regression. Sensitivity analysis was performed to evaluate the extent to which inferences might depend on a particular study. Publication bias was assessed by visual inspection of funnel plots. Formal statistical assessment of funnel plot asymmetry was done with Begg’s and Egger’s regression asymmetry test. Statistical analyses were carried out by the use of Stata, version 11.2 (Stata Corp, College Station, TX). P values that were less than 0.05 were considered as statistically significant.

### Consent for publication

All authors approved the final manuscript for submission and publication.

## Results

### Study characteristics

**Figure 1:**
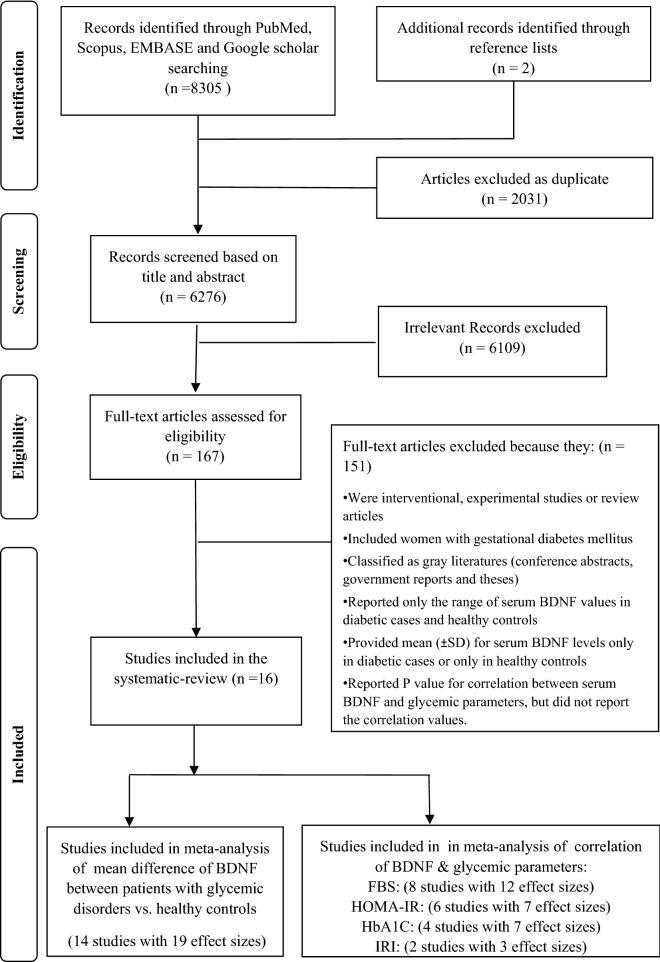
Flowchart diagram describing the process of study selection.

As shown in Fig. 1, the primary search of main databases resulted in 8305 studies. After eliminating duplicates, 6276 records remained. Titles and abstracts were screened and irrelevant articles were excluded. Of those studies, the full-text of 167 articles was assessed with details for the fulfillment of the inclusion criteria of the present review. Finally, 16 studies^7,20–25,27–35^ were included in the present systematic review and meta-analysis. Fourteen studies have reported the average serum/plasma BDNF values patients with glycemic disorders vs. healthy controls^7,20–25,27–30,32,33,35^. Eight articles have depicted the correlation between FBG and serum/plasma BDNF levels^7,20,23–25,29,31,35^; 6 investigations have explored the correlation between HOMA-IR and BDNF concentrations^7,20,22–25^. Also, 4 and 2 studies have respectively reported the correlation between serum/plasma BDNF levels with HbA1c and immunoreactive insulin (IRI)^7,23,25,34^.

Characteristics of 16 eligible articles are identified in Table 1. These investigations were conducted between 2006 and 2020. Ten of them were performed in Asian countries and the others were conducted in non-Asian countries. In case of study design, 13 studies had case–control and 3 others had cross-sectional design. The number of participants varied from 31 to 492 with a mean age ranged between 37 and 66.3 years old. Three included studies were conducted on female subjects and 13 others were performed among both sexes. Participants in case groups were patients with T2D or patients suffered from other glycemic disorders including impaired glucose tolerance (IGT), impaired insulin function and metabolic syndrome. BDNF levels were assessed in serum in 13 eligible studies; while 3 others used plasma samples. Blood samples were collected after an overnight fast in all investigations, except in one study which were gathered in a preoperative state. Mean BDNF levels ranged from 0.37 to 31 (ng/mL) in cases with T2D or other glycemic disorders and 0.13 to 39 (ng/mL) in healthy controls. Regarding the correlation test, 5 studies have used a Pearson test, and 2 studies have used partial correlation analysis, while 3 others have used a Spearman correlation test. Correlation values between FBG and serum/plasma BDNF levels ranged from − 0.320 to 0.754. With regard to quality score of included studies in the analysis of mean BDNF values in cases vs. controls, the median score was 5; while the median score of studies which reported the correlation between circulating BDNF concentrations and glycemic parameters was 7 (Supplemental Tables 1 and 2).

**Table 1:** Characteristics of the studies that were included in the present systematic review and meta-analysis.

First author, year	Country	Type of study	Subjects (case/control)	Subjects (M/F)	Mean age (yr)	Health status	Mean BDNF ± SD (case/control) ng/mL	Sample/participants condition	Duration of T2D (mean ± SD or med (IQR))	Type of correlation	Correlation (health status/glycemic parameter: r)	Study quality score^a^
Boyuk 2014	Turkey	Case–control	88/33	55/66	60.3	T2DM/healthy	0.2068 ± 0.1073/0.130 ± 0.059	Serum/fasting state	4.03 ± 3.38	Spearman	T2DM/HOMA-IR: 0.281	6
Portillan 2019	Arizona	Cross-sectional	0/349	116/233	41.6	Healthy	33 ± 16	Serum/fasting state	–	Pearson	Healthy/2hPG: 0.10, FBG: 0.11	6
Fujinami 2008	Japan	Case–control	112/80	91/80	57.8	T2DM/healthy	15.5 ± 5.2/20.0 ± 7.3	Serum/fasting state	8.64 ± 7.38	Spearman	Male: healthy/HbA1c: 0.015, FBG: 0.101, T2DM/HOMA-IR: 0.206, HbA1c: 0.059, FBG: − 0.112, IRI: 0.205 Female: Healthy/HbA1c: − 0.065, FBG: − 0.020 T2DM/HOMA− IR: 0.44, HbA1c: − 0.133, FBG: − 0.105, IRI: 0.458	7
Sun 2018	China	Case–control	83/110	88/105	57.3	T2DM/diabetic peripheral neuropathy/healthy	21.26 ± 17.11/17.39 ± 13.2/22.46 ± 3.31	Serum/fasting state	NR	–	–	3
Suwa 2006	Japan	Case–control	24/7	0/31	49.3	T2DM/healthy	40.6 ± 9.9/30.6 ± 7.2	Serum/fasting state	Newly diagnosed	Pearson	T2DM/FBG: 0.437, HOMA-IR: 0.506, HbA1c: 0.397, IRI: 0.342, healthy/FBG: 0.754	6
Jabbari 2014	Iran	Case–control	43/43	0/86	37	Premenopausal women with MetS/healthy premenopausal women	6.83 ± 2.20/8.59 ± 2.10	Serum/fasting state	NR	Pearson	Metabolic syndrome/FBG: − 0.320, HOMA-IR: − 0.390	4
Li 2016	China	Case–control	292/200	156/244	60	T2DM/healthy	15.9 (12.6–19.8)/24.6 (17.2–27.8) Median (IQR)	Serum/fasting state	8.5 (5.0–13.5)	Spearman	T2DM/FBG: − 0.394, HOMA-IR: − 0.406	6
Uzel 2020	Turkey	Case–control	31/37	38/30	66.3	Diabetic/diabetic with non-PDR/diabetic with PDR/healthy	0.372 (0.307–0.450)/0.272 (0.2–0.389) 0.187 (0.104–0.59) 0.483 (0.13–0.919)/median (IQR)	Serum/not declared; preoperative state	10.67 ± 8.21/14 ± 4.42/15.31 ± 7.58	–	–	6
Wei 2015	China	Cross-sectional	92/81	92/81	61	T2DM/normal glucose tolerance (NGT)	1.77 (1.13–2.17)/1.32 (1.13–1.57) Median (IQR)	Serum/fasting state	Newly diagnosed	Partial correlation	T2DM and healthy/FBG: 0.206, HOMA-IR: 0.211, PPG: 0.076, HbA1c: 0.146	9
Zheng 2018	China	Cross-sectional	1833/0	843/990	66.3	T2DM	1.685 ± 0.590	Plasma/fasting state	9.4 ± 7.3	Partial correlation	T2DM/HbA1c :0.005	9
Krabbe 2007	Denmark	Case–control	34/96/103	164/69	60.6	IGT/T2DM/NGT	1.915 (0.925)/1.563 (0.888)/2.364 (1.405)	Plasma/fasting state	NR	Pearson	T2DM/FBG: − 0.300	5
Arentoft 2009	USA	Case–control	41/41	40/42	61.5	Impaired insulin function/healthy	3.85 ± 1.32/4.56 ± 1.6	Plasma/fasting state	NR	–	–	3
Ola 2012	Saudi Arabia	Case–control	69/19	Not reported	51.8	T2DM/diabetic with PDR/healthy	21.8 ± 4.9/10.01 ± 8.1/25.5 ± 8.5	Serum/fasting state	NR	–	–	5
Ortiz 2016	Mexico	Case–control	37/40	0/77	46.3	T2DM/healthy	31.55 ± 10.24/39.36 ± 8.9	Serum/fasting state	14.3 ± 6.22	–	–	3
Zhen 2013	China	Case–control	208/212	178/242	51.1	T2DM/healthy	8 ± 2.6/11.9 ± 2.6	Serum/fasting state	6.1 ± 1.1	Pearson	T2DM and healthy/FBG: − 0.180	8
He 2014	China	Case–control	37/37	50/24	55.1	T2DM/healthy	22.04 ± 6.72/28.53 ± 16.14	Serum/fasting state	7.05 ± 5.46	–	–	1

*M* male, *F* female, *BDNF* brain-derived neurotropic factor, *ng/mL* nano-gram/milli-liter, *SD* standard deviation, *IQR* inter-quartile range, *T2DM* type 2 diabetes mellitus, *FBG* fasting blood glucose, *HOMA-IR* homeostatic model assessment for insulin resistance, *HbA1c* glycosylated hemoglobin, *IRI* immunoreactive insulin, *PDR* proliferative diabetic retinopathy, *NR* not reported.

^a^Based on Newcastle–Ottawa Scale (NOS).

### Finding from meta-analysis of mean difference of serum/plasma BDNF levels in patients with T2D vs. healthy controls

**Figure 2:**
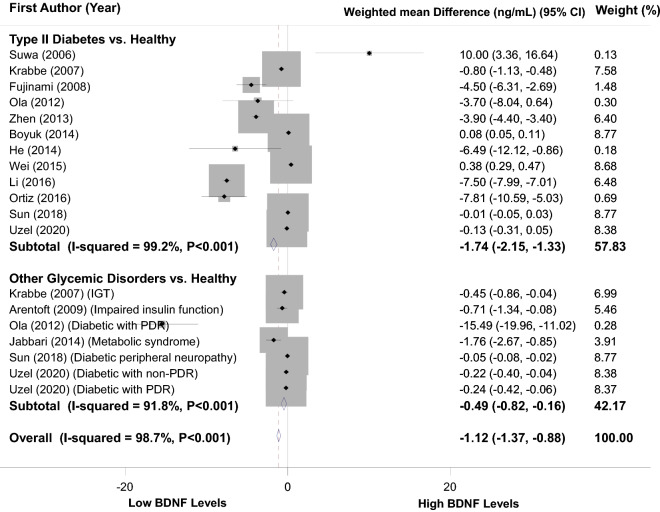
Forest plots depicting the weighted mean difference of serum/plasma BDNF levels of patients with Type 2 Diabetes or other glycemic disorders compared to healthy controls.

As clarified in Fig. 2, 19 size effects from 14 studies^7,20–25,27–30,32,33,35^ with a total of 1306 diabetic or other glycemic disorders cases vs. 1250 healthy controls were included in this analysis. Overall weighted mean difference (WMD) of BDNF levels in patients with T2D (or other glycemic disorders) was 1.12 ng/mL lower than healthy subjects (WMD: − 1.12; 95%CI − 1.37, − 0.88), which was statistically significant. Heterogeneity between included studies was observed (I^2^ = 98.7%, P < 0.001); therefore, subgroup analysis based on the type of glycemic disorders was done (Fig. 2). In patients with T2D, BDNF levels were lower than healthy controls (WMD: − 1.74 ng/mL; 95%CI − 2.15, − 1.33). Also, subjects with other glycemic disorders had lower blood BDNF values than healthy controls (WMD: − 0.49 ng/mL; 95%CI − 0.82, − 0.16); however, significant heterogeneity was found in both subgroups (I^2^ = 99.2%, P < 0.001 and I^2^ = 91.8%, P < 0.001, respectively).

Subgroup analyses based on other variables [including BDNF matrices (serum vs. plasma), Asian vs. non-Asian countries, age of ≥ 60 vs. < 60, and high quality vs. low quality] were conducted and the findings are reported in Table 2. These stratified analyses could not significantly explain the between-study heterogeneity. After excluding each included study, no significant difference in heterogeneity was observed. Furthermore, meta-regression analysis based on mean age of participants was conducted; but heterogeneity remained unchanged (β = 0.078, P = 0.46, I^2^ residual = 98.77%, Adjusted r^2^ = 1.44%). Meta-regression based on mean duration of diabetes (reported only in 9 of 14 studies) showed that this variable did not significantly related to serum/plasma BDNF values in cases vs. controls (β = − 0.162, P = 0.64, I^2^ residual = 99.38%, Adjusted r^2^ = − 10.72%) and could not decrease the observed heterogeneity. Sensitivity analysis was performed; exclusion of each included investigation did not significantly change the overall effect, except one; after excluding the study of Li^20^ the finding was relatively shifted toward null, but was still significant (WMD: − 0.47 ng/mL; 95%CI − 0.63, − 0.31). Publication bias was also assessed; there was no asymmetry in funnel plot and no publication bias was found according to the Begg’s test (P = 0.15).

**Table 2:** Findings from subgroup analysis of mean difference of serum BDNF levels in cases with T2D or glycemic disorders vs. healthy controls as well as correlation between serum BDNF levels and glycemic parameters.

	No. of effect sizes	Fisher’s Z (95% CI)	P within^a^	I^2^ (%)	P between^b^
**Subgroup analysis for mean difference of serum BDNF levels in cases with T2D or glycemic disorders vs. healthy controls**					
Overall	19	− 1.12 (− 1.37, − 0.88)	< 0.001	98.7	
BDNF matrices					< 0.001
Serum	16	− 1.24 (− 1.51, − 0.97)	< 0.001	98.9	
Plasma	3	− 0.67 (− 0.91, − 0.44)	0.418	00.0	
Asian vs. Non-Asian					< 0.001
Asian	9	− 2.05 (− 2.53, − 1.58)	< 0.001	99.4	
Non-Asian	10	− 0.50 (− 0.82, − 0.18)	< 0.001	93.7	
Age ≥ 60 vs. < 60					< 0.001
≥ 60	8	− 1.04 (− 1.61, − 0.47)	< 0.001	99.3	
< 60	11	− 1.29 (− 1.62, − 0.96)	< 0.001	97.4	
Quality score^c^					< 0.001
Low quality (scores ≤ 5)	8	− 1.77(− 2.43, − 1.11)	< 0.001	99.4	
High quality (scores > 5)	11	− 0.43(− 0.64, − 0.22)	< 0.001	92.8	
**Subgroup analysis for correlation between BDNF and FBG**					
Overall	12	− 0.05 (− 0.21, 0.11)	< 0.001	86.3	
BDNF matrices					0.02
Serum	11	− 0.02 (− 0.19, 0.15)	< 0.001	86.7	
Plasma	1	− 0.31 (− 0.50, − 0.12)	–	–	
Asian vs. Non-Asian					0.02
Asian	11	− 0.02 (− 0.19, 0.15)	< 0.001	86.7	
Non-Asian	1	− 0.31 (− 0.50, − 0.12)	–	–	
Age ≥ 60 vs. < 60					0.02
≥ 60	2	− 0.11 (− 0.72, 0.51)	< 0.001	97.6	
< 60	10	− 0.05 (− 0.19, 0.10)	< 0.001	72.7	
Quality score^c^					0.02
Low quality (scores < 7)	6	− 0.06 (− 0.34, 0.23)	< 0.001	91.6	
High quality (scores ≥ 7)	6	− 0.02 (− 0.18, 0.14)	< 0.001	68.0	
**Subgroup analysis for correlation between BDNF and HOMA-IR**					
Overall	7	0.12 (− 0.20, 0.44)	< 0.001	93.7	
Asian vs. Non-Asian					0.001
Asian	6	0.09 (− 0.27, 0.45)	< 0.001	94.1	
Non-Asian	1	0.29 (0.08, 0.50)	–	–	
Age ≥ 60 vs. < 60					< 0.001
≥ 60	3	0.02 (− 0.47, 0.51)	< 0.001	96.7	
< 60	4	0.20 (− 0.22, 0.62)	< 0.001	86.2	
Quality score^c^					< 0.001
Low quality (scores < 7)	4	− 0.02 (− 0.49, 0.45)	< 0.001	93.9	
High quality (scores ≥ 7)	3	0.28 (0.12, 0.44)	0.223	33.3	

^a^P for heterogeneity, within subgroup.

^b^P for heterogeneity, between subgroups.

^c^Quality Scores were according to Newcastle–Ottawa Scale.

### Finding from meta-analysis of correlation between serum/plasma BDNF levels and FBG

**Figure 3:**
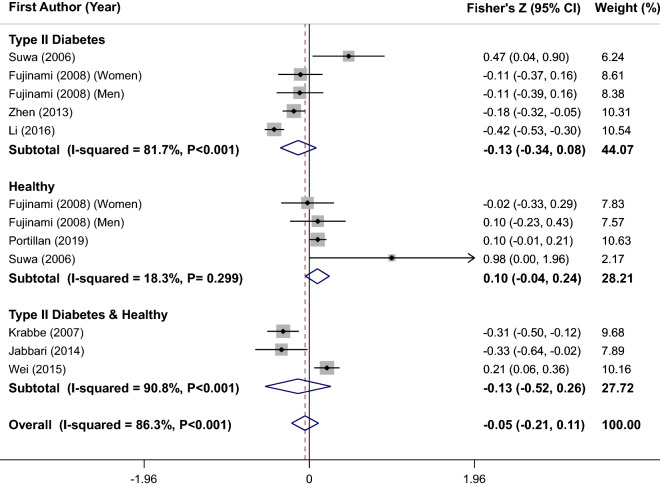
Forest plot of correlation between serum/plasma BDNF levels and fasting blood glucose (FBG); stratified by health status of participants.

Combining 12 effect sizes from 8 studies^7,20,23–25,29,31,35^ included 1400 participants showed that the overall correlation between BDNF levels and FBG was not statistically significant (overall Fisher’s Z = 0.05; 95%CI − 0.21, 0.11), as shown in Fig. 3. Between studies heterogeneity was significant (I^2^ = 86.3%, P < 0.001); so, stratified analysis based on health status of participants was performed. In studies on healthy subjects, there was no heterogeneity (I^2^ = 18.3%, P = 0.30); but in subgroup of individuals with T2D (I^2^ = 81.7%, P < 0.001) and subgroup of both healthy and those with T2D or other glycemic disorders (I^2^ = 90.8%, P < 0.001), heterogeneity was observed (Fig. 3). Therefore, subgroup analyses based of other covariates were performed and findings are reported in Table 2. Stratified analyses according to BDNF matrices (serum vs. plasma), study location, age and quality score of studies could not completely explain the observed heterogeneity. Furthermore, sensitivity analysis was conducted and after removing of each study at a time, no significant change was showed. No evidence of publication bias was also found (Begg’s test = 0.22 and Egger’s test = 0.48).

### Finding from meta-analysis of correlation between serum BDNF levels and HOMA-IR

**Figure 4:**
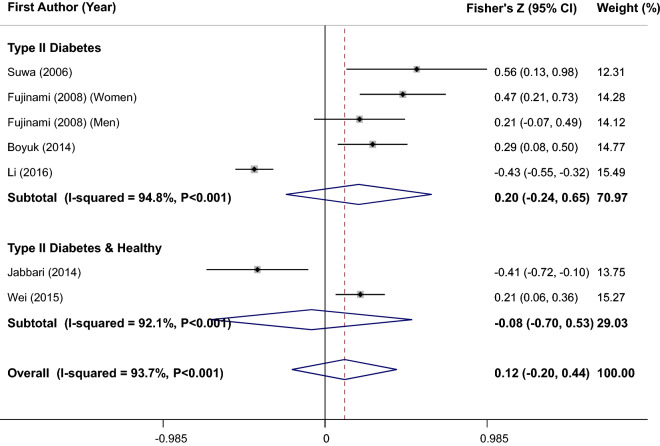
Forest plot of correlation between serum BDNF levels and Homeostatic Model Assessment for Insulin Resistance (HOMA-IR); stratified by health status of participants.

Combining 7 size effects from 6 studies^7,20,22–25^ with 732 participants resulted in a non-statistically significant overall correlation between serum BDNF levels and HOMA-IR (overall Fisher’s Z = 0.12; 95%CI − 0.2, 0.44) (I^2^ = 93.7%, P < 0.001) (Fig. 4). In order to find the source of observed between-studies heterogeneity, subgroup analyses based on health status of participants were performed (Fig. 4); but heterogeneity remained significant in subgroups. Stratified analyses based on Asian vs. non-Asian societies, age and quality score were carried out and suggested no significant difference in findings or explanation for heterogeneity between studies (Table 2). In addition, sensitivity analysis was performed and revealed that eliminating of each included study made no change in results. Also, publication bias was tested and no evidence of bias was found (Begg’s test = 0.88 and Egger’s test = 0.17).

### Finding from meta-analysis of correlation between serum/plasma BDNF levels and HA1c

**Figure 5:**
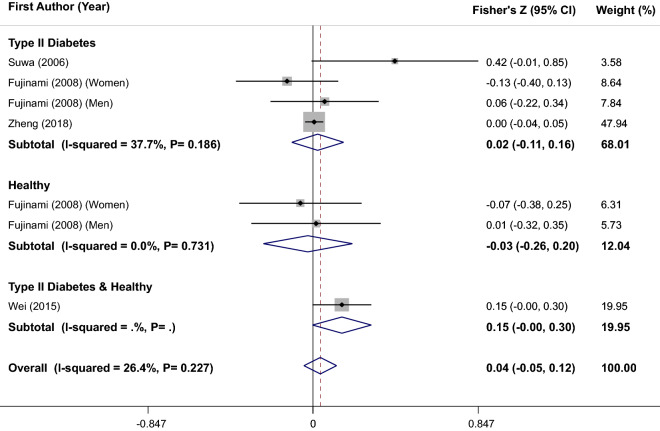
Forest plot of correlation between serum/plasma BDNF levels and glycated haemoglobin (HbA1C); stratified by health status of participants.

Data from 7 size effects of 4 studies^7,23,25,34^ included 2222 participants were combined and no significant correlation was found (overall Fisher’s Z = 0.04; 95%CI − 0.05, 0.12) (Fig. 5), without between-study heterogeneity (I^2^ = 26.4%, P = 0.23). Stratified analysis showed that the correlation was not significant in both T2D patients and healthy subjects (Fig. 5). Excluding the study by Zheng et al.^34^, which measured BDNF values in plasma, did not alter the finding (Fisher’s Z = 0.06; 95%CI − 0.06, 0.19, I^2^ = 25.5%, P = 0.24). Results from sensitivity analysis revealed no significant change in overall estimate by excluding each study. Also, no evidence of publication bias was found (Begg’s test = 0.18 and Egger’s test = 0.49).

### Finding from meta-analysis of correlation between serum BDNF levels and immunoreactive insulin (IRI)

Pooling 3 correlations from 2 studies^23,25^ included 136 participants showed that serum BDNF values were positively correlated with IRI (overall Fisher’s Z = 0.36; 95%CI 0.18, 0.54). No evidence of between-study heterogeneity was found (I^2^ = 8%, P = 0.34) (data not shown).

## Discussion

We found that serum/plasma BDNF levels in patients with T2D or individuals with other glycemic disorders were significantly lower than healthy controls. No significant correlation between BDNF concentrations and glycemic parameters including (FBG, HOMA-IR and HbA1c) was found, while pooling estimates showed a positive significant correlation between serum BDNF levels and immunoreactive insulin values.

Type 2 diabetes as an inflammatory condition is associated with the failure of kidneys, heart and blood vessels^1,19^. Our findings showed that diabetic patients had lower serum/plasma BDNF levels than healthy ones. In experimental studies, treatment with BDNF could prevent the development of diabetes in prediabetic db/db mice^36^. Also, BDNF reduces serum insulin and glucose levels when injected into obese diabetic mice^37^. These results suggested that BDNF might have a role in the treatment of diabetes. Improvement of serum BDNF levels might decrease the risk of T2D and its complications, as well. Also, T2D could elevate the incidence risk of depression^38^ and cognitive impairment and dementia^39^. The connecting link between depression and T2DM might be BDNF. Depression is a well-known risk factor for the development of T2DM, whereas most diabetic patients also have depression. So, enhanced levels of BDNF might decrease the prevalence of these disorders.

Several pervious investigations have evaluated the relationship between serum/plasma BDNF levels and non-communicable diseases (NCDs). In a recently published meta-analysis of observational studies, serum BDNF levels in patients with Alzheimer’s disease (AD) and individuals with mild cognitive impairment (MCI) in comparison to healthy controls have been examined and the results showed a reduced serum BDNF level in patients with AD compared to healthy subjects; but no significant relationship between MCI and serum BDNF levels was found^40^. Another meta-analysis has confirmed that patients with Anorexia-nervosa (AN) had significantly lower circulating BDNF concentrations than healthy controls^41^. Also, investigating 52 cross-sectional and longitudinal studies in a meta-analysis has documented a decreased level of BDNF, more clearly in plasma BDNF than in serum, in manic and depressive cases vs. healthy controls^42^. However, a meta-analysis of 10 studies with regard to the association between circulating BDNF levels and obesity showed that obese patients had levels of BDNF similar to controls with normal-weight^43^. The small sample size in the mentioned meta-analysis (307 obese patients and 236 controls) could lead to a non-significant relationship. Moreover, the correlation between plasma BDNF and inflammatory markers such as CRP in T2D patients was investigated and no significant relationship was observed^29^. In the present study, reduced levels of BDNF in diabetic patients and in those who suffered from other glycemic disorders vs. healthy controls was found. Different findings might be due to different study design, sample sizes, health or disease status of participants or other differences in investigations. For instance, in the current meta-analysis no cohort study could be included, while some above-mentioned meta-analyses included cohort and longitudinal studies along with case–control and cross-sectional reports^42,43^.

Previous investigations have documented that BDNF levels could vary according to its source, meaning plasma versus serum values^42,43^. In the current meta-analysis, most of included studies have used serum BDNF levels, with far less studies conducted on plasma. In fact, BDNF concentrations in serum are 20- to 50-times higher than in plasm^43^. So, serum and plasma represent two different compartments. Platelets are the high storage of peripheral BDNF, can actively absorb it from the circulation, and release it in serum during the clotting process. Thus, serum BDNF levels largely reflect the pool of BDNF stored and released from platelets during coagulation, in contrast to plasma BDNF values^8^. We did not find a significant difference among serum and plasma BDNF in T2D/glycemic disorders, compared to healthy individuals. However, our results might be influenced by potential limitations that are herein critically discussed. Circulating BDNF levels could change within a day. After BDNF production by the brain, it is released into the blood circulation; but it has a half-life of less than 10 min and is rapidly cleared from the blood mostly by the liver, in contrast to a platelet’s life-span of 9–11 days^44^. So, a single blood draw might not be representative of a potential relationship between glycemic control and BDNF, because it could not capture rising and falling of BDNF throughout the day. In addition, blood BDNF concentrations could be influenced by environmental factors and might well reflect the physiological conditions of the body such as inflammation, exercise activity, and eating/fasting^8^. Physical exercise could improve serum BDNF in diabetic patients^45^. Moreover, blood samples were collected after an overnight fast in all included investigations, except in one study^21^ which were gathered in a preoperative state. However, blood samples were not collected at the same time of the morning or the similar hours of fasting. It is also worth noting that the included studies did not apply the same procedures of sampling, handling, and storage; these differences made it difficult to critically analyze the provided data. Furthermore, it has been reported that BDNF levels differ between men and women in both plasma and serum^46^. So, the different percentage of women in the studies could influence the results. Matching by sex and age or adjustment for these confounders is particularly crucial in case–control studies with few participants^46^; but this issue was not considered in some included studies. These limitations should be carefully considered for future studies on BDNF, and more efforts should be made to reduce the impact of these limitations.

Several potential mechanisms could link BDNF to development of T2D. First, experimental studies revealed that BDNF involves in pathobiology of T2D by modulation of secretion and actions of insulin, glucagon, leptin, ghrelin, various neurotransmitters and peptides, and pro-inflammatory cytokines related to energy homeostasis^25^. Second, serum BDNF levels showed a negative correlation with inflammation and hs-CRP concentration in individuals with T2D^20^. An increase in BDNF levels might be associated with protecting neurons from inflammatory injury in diabetic patients, since inflammatory processes in CNS are considered neurotoxic^47^. During a CNS injury, immune system cells synthesize and secrete a variety of neurotrophins including nerve growth factor (NGF) and BDNF, which could lead to expression of tyrosine kinase (Trk) receptor family such as TrkA, TrkB and TrkC. This process could result in a variety of modulations of immune and neuronal cell function^47^. In fact, it has been suggested that BDNF, as an endogenous cytoprotective molecule, could protect cells against stress and inflammation, restore anti-oxidant defenses to normal values and thus, prevent apoptosis, preserve insulin secreting capacity of β cells and prevent development of T2D^48–50^. Third, a decrease in BDNF might contribute in pathogenesis of T2D, because it has been suggested that BDNF might have anti-diabetic effects. A recent experimental study on diabetic mice showed that the brain intraventricular administration of BDNF mitigated diabetic hyperglycemia through an insulin-independent mechanism involving inhibition of glucagon secretion and decrease in hepatic glucose production^51^. In vivo administration of BDNF to normal mice (10 mg/kg/day) for 7 days has also resulted in a decreased secretion of glucagon^52^. Furthermore, Meek et al. found that repeated daily intracerebroventricular administration of BDNF attenuated diabetic hyperglycemia independent of changes in ingested food. Instead, BDNF could lower blood glucose levels by suppressing hepatic glucose production, without changes in tissue glucose uptake, and via normalizing both plasma glucagon levels and hepatic expression of gluconeogenic genes^53^. In this way, a sort of positive feedback relationship might be hypothesized between BDNF and T2D.

Previous investigations have shown that BDNF and insulin act in combination, not only in CNS, but also in the peripheral nervous system and peripheral tissues. Insulin could act as an adiposity signal to the brain by acting on the arcuate nucleus (ARC) of the hypothalamus which controls energy homeostasis^54^. On the other hand, BDNF could have a protective effect on pancreatic islets in obese diabetic mice^37^. BDNF could prevent exhaustion of the pancreas in diabetic mice by maintaining the histologic cellular organization of beta cells and non-beta cells in pancreatic islets and restoring the level of insulin-secreting granules in beta cells^37^. BDNF, insulin and insulin-like growth factor (IGF)-1 protect hippocampal neurons from serum deprivation-induced death by activating the PI3K/Akt pathway^55^, while BDNF accelerates insulin-stimulated activation of PI3K in peripheral tissues, including skeletal muscle, brown adipose tissue and the liver^56^. Animal studies have demonstrated that BDNF treatment in diabetic mice could significantly suppress the blood glucose, food consumption, and weight gain by enhancing energy expenditure, glucose and lipid metabolism^57^. Once or twice per week BDNF administration (70 mg/kg/wk) to db/db mice for 3 weeks could significantly reduce FBG and HbA1c. These results suggested that BDNF could be a novel hypoglycemic agent even with treatment as infrequently as once per week^58^. However, the therapeutic potential of BDNF is restricted due to its short half-life (< 10 min) and inability to cross the blood–brain barrier (BBB) because of its large size (27 kDa). So, some small molecules, such as 7,8-dihydroxyflavone (7,8-DHF), that could mimic BDNF, for non-invasive clinical application. 7,8-DHF would have a longer half-life and capability of penetrating the blood–brain barrier. Also, following intravenous injection of nanoparticle-bounded BDNF, increased BDNF levels were found in the brain, and neurological and cognitive functions were improved in animal studies^59^. Moreover, intraportal administration of Glucagon-like peptide (GLP)-1 could increase the BDNF content in the pancreas and decrease glucagon secretion, as well as blood glucose levels^60^. Using co-factors such as vitamin B_12_ and dexamethasone could also up-regulate the expression of BDNF, and might be attempted while pursuing the clinical applications of BDNF^58^.

BDNF is widely expressed not only in the CNS, but also in gut, differentiated human muscle cells and other tissues, binds to its high affinity receptor TrkB and activates signal transduction cascades (IRS1/2, PI3K, Akt), that can lead to encode proteins involved in beta cell survival^61,62^. BDNF regulates functions of the gut and pancreatic β-islet activity in response to plasma levels of glucose, protein, fatty acids, insulin, and leptin. Thus, gut acts as a neuroendocrine organ, responds rapidly to ingested food and influences the satiety and metabolism by producing factors such as leptin, cholecystokinin (CCK) and BDNF, releases incretins that enhance insulin secretion from pancreatic β-cells, and sends messages to the brain by the intestine–vagus pathway. Then, acetylcholine, as a vagal mediator, and possibly BDNF could modulate the secretion and actions of various hypothalamic neurotransmitters and peptides, and, in this gut–brain–liver axis, hypothalamus integrates all the messages received from the gut to regulate plasma glucose levels^61^. On the other hand, muscle-derived BDNF might be a key factor mediating increased glucose metabolism in response to exercise, with implications for the treatment of T2D^62^.

Newly diagnosed patients with T2D had significantly higher serum BDNF levels than healthy controls in some previous investigations^22,23^; such that it was suggested that serum BDNF could be used as a predictive biomarker for T2D like HgA1c in future^22^. Interestingly, other studies found that BDNF was negatively associated both T2D duration and FBS levels^20,25,29,35^, suggesting that the decreased BDNF levels in T2D might be related to longer diabetes duration and higher glucose concentrations. These findings indicated that patients with long-standing T2D might have lower serum BDNF levels. So, it could be speculated that lower BDNF levels appear to occur later in T2D^25,35^. Reduced BDNF levels might be a predictor for T2D complications after an initial rise in BDNF as the brain initially attempts to adjust to the metabolic insult of T2D through increasing BDNF production^20,25^. Therefore, the inconsistencies between investigations might be explained, in part, by the duration of T2D. Unfortunately, some of included investigations in the current analysis did not provide information of duration of T2D or included participants with a wide range of T2D duration; thus, we could not consider this critical covariate in the sub-group analysis. Taken together, these findings suggest that future studies should consider T2D duration while assessing the relationship or correlation between serum BDNF levels with T2D or glycemic profiles.

It is not clearly known if a decrease in serum BDNF level in diabetic patients is a cause and pre-requisite for the occurrence of glycemic disorder or a consequence of T2D or elevated glucose levels, since high levels of glucose, but not insulin, could prevent the output of BDNF from the brain^58^. The precise mechanism of decreased BDNF levels in diabetic patients remained unknown. One factor which could affect the BDNF circulating levels in diabetic patients vs. healthy controls might be different acute and chronic stress response in these patients which could influence the release of BDNF^21,63^. It was also suggested that obese individuals had lower BDNF levels. As most patients with type 2 diabetes are obese, they could have reduced BDNF levels^28^. Furthermore, high plasma glucose levels or hyperglycaemia could negatively influence BDNF output from the brain^20,29^. Another possible explanation is that circulating levels of BDNF can be affected by lifestyle factors such as physical inactivity and unhealthy diet, which are common risk factors of T2D^31^.

The current meta-analysis has some strengths and inherent limitations. This was the first systematic review and meta-analysis that assessed BDNF levels in diabetic cases vs. healthy controls. Also, we evaluated the relationship between glycemic parameters and BDNF levels. Moreover, due to meta-analysis design, we could find overall effect based on each study weight. However, all included investigations had either a case–control or cross-sectional design. Another limitation was the small total number of conducted studies. Moreover, the overall effect size was much higher in lower quality studies than high quality ones. Lower quality studies were those that did not clearly define the control group or cases, included volunteers as the controls, and did not consider the most important confounders^46^, such as age and sex in their design or analysis. So, the possibility that selection bias might influence the results cannot be ruled out. Also, included studies had small worldwide distribution. Furthermore, the relationship between BDNF and glycemic parameters was considered as a secondary goal in some eligible studies; sample sizes were not calculated for secondary outcomes and the small sample sizes of studies should be considered when explaining our results. Also, some of studies measured BDNF levels only in diabetic cases and not in controls. So, we could not include them in the current analysis. Finally, we could not perform gender-stratified analysis, since the included studies did not provide separate reports for men and women. Also, it remains to be explored that administration of BDNF could be applied as a new therapeutic approach in both prevention and management of T2D.

In conclusion, we found that diabetic patients and subjects with glycemic disorders had lower circulating BDNF levels than healthy controls. However, there was no significant correlation between BDNF concentrations and glycemic parameters (including FBG, HOMA-IR and HbA1c). Further investigations, especially with prospective design, are required to confirm these findings. In addition, a better understanding of the role of this endogenous peptide in health and disease may pave the way to exploit BDNF as a novel therapeutic agent for neurodegenerative and metabolic diseases such as T2D.

## Supplementary Information

Supplementary Information 1.

Supplementary Information 2.

## Abbreviations

BDNF: Brain-derived neurotrophic factor
FBG: Fasting blood glucose
HOMA-IR: Homeostatic model assessment for insulin resistance
HbA1c: Glycosylated hemoglobin
IRI: Immunoreactive insulin
T2D: Type 2 diabetes
95%CI: 95% confidence intervals
PRISMA: Preferred Reporting Items for Systematic Reviews and Meta-Analyses guideline
NOS: Newcastle–Ottawa Scale
SD: Standard deviation
SE: Standard error
WMD: Weighted mean difference
OGTT: Oral glucose tolerance test
IR: Insulin resistance
MetS: Metabolic syndrome
CNS: Central nervous system
NCDs: Non-communicable diseases
AD: Alzheimer’s disease
MCI: Mild cognitive impairment
AN: Anorexia-nervosa
NR: Not reported
