# Supplementary material for: A systematic review and meta-analysis of association between brain-derived neurotrophic factor and type 2 diabetes and glycemic profile
Source: Sci Rep. 2021 Jul 2;11:13773. doi: 10.1038/s41598-021-93271-z (PMC8253793; doi:10.1038/s41598-021-93271-z)
Supplement: Supplementary file 2 — Supplementary Information 2. [file 41598_2021_93271_MOESM2_ESM.docx]

| **Supplemental Table 1.** Details of quality assessment of included case-control studies in the systematic review and meta-analysis based on Newcastle-Ottawa Scale^1^. | | | | | | | | |
| --- | --- | --- | --- | --- | --- | --- | --- | --- |
|  | Is the case definition adequate?    Representativeness of the sample    Representativeness of the sample | Representativeness of the cases | Selection of controls | Definition of controls | Comparability of cases and controls on the basis of the design or analysis | Ascertainment of exposure | Non-response rate | Total score |
| Suwa et al, 2006 | ***** | ***** |  |  | ****** | ***** | ***** | 6 |
| Krabbe et al, 2007 | * |  | ***** | * |  | * | * | 5 |
| Fujinami et al, 2008 | * | * | * |  | ** | * | * | 7 |
| Ola et al, 2012 | ***** | ***** |  | ***** |  | * | * | 5 |
| Zhen et al, 2013 | ***** | ***** | ***** | ***** | ****** | ***** | ***** | 8 |
| Boyuk et al, 2014 | ***** | ***** |  |  | ****** | ***** | ***** | 6 |
| He et al, 2014 |  |  |  |  |  | ***** |  | 1 |
| Li et al, 2016 |  | ***** | ***** |  | ****** | ***** | ***** | 6 |
| Ortiz et al, 2016 |  |  | ***** |  |  | ***** | ***** | 3 |
| Sun et al, 2018 |  | ***** |  |  |  | ***** | ***** | 3 |
| Uzel et al, 2020 | ***** | ***** |  |  | ****** | ***** | ***** | 6 |
| Arentoft et al, 2009 |  |  |  |  | ****** |  | ***** | 3 |
| Jabbari et al, 2014 | ***** |  |  |  | ***** | ***** | ***** | 4 |

| **Supplemental Table 2.** Details of quality assessment of included cross-sectional studies in the systematic review and meta-analysis based on Newcastle-Ottawa Scale^1^. | | | | | | | | |
| --- | --- | --- | --- | --- | --- | --- | --- | --- |
|  | Representativeness of the sample    Representativeness of the sample    Representativeness of the sample    Representativeness of the sample | Sample size | Non-respondents | Ascertainment of the exposure (risk factor) | Comparability of subjects in different outcome groups | Assessment of outcome | Statistical test | Total score |
| Wei et al, 2015 | ***** | ***** |  | ****** | ****** | ****** | ***** | 9 |
| Portillan et al, 2019 | ***** | ***** |  |  | ***** | ****** | ***** | 6 |
| Zheng et al, 2018 | ***** | ***** |  | ****** | ****** | ****** | ***** | 9 |

1Wells GA, Shea B, O’Connell D, Peterson J, Welch V, Tugwell P. The Newcastle-Ottawa Scale (NOS) for Assessing the Quality of Nonrandomised Studies in Meta-Analyses. Available from: http://www.ohri.ca/programs/clinical_epidemiology/oxford.asp
